# The Variable Vector Countermeasure Suit (V2Suit) for space habitation and exploration

**DOI:** 10.3389/fnsys.2015.00055

**Published:** 2015-04-10

**Authors:** Kevin R. Duda, Rebecca A. Vasquez, Akil J. Middleton, Mitchell L. Hansberry, Dava J. Newman, Shane E. Jacobs, John J. West

**Affiliations:** ^1^The Charles Stark Draper Laboratory, Inc.Cambridge, MA, USA; ^2^Massachusetts Institute of TechnologyCambridge, MA, USA; ^3^David Clark Company, Inc.Worcester, MA, USA

**Keywords:** human spaceflight, biomechanics, control moment gyroscope, wearable electronics, countermeasures

## Abstract

The “Variable Vector Countermeasure Suit (V2Suit) for Space Habitation and Exploration” is a novel system concept that provides a platform for integrating sensors and actuators with daily astronaut intravehicular activities to improve health and performance, while reducing the mass and volume of the physiologic adaptation countermeasure systems, as well as the required exercise time during long-duration space exploration missions. The V2Suit system leverages wearable kinematic monitoring technology and uses inertial measurement units (IMUs) and control moment gyroscopes (CMGs) within miniaturized modules placed on body segments to provide a “viscous resistance” during movements against a specified direction of “down”—initially as a countermeasure to the sensorimotor adaptation performance decrements that manifest themselves while living and working in microgravity and during gravitational transitions during long-duration spaceflight, including post-flight recovery and rehabilitation. Several aspects of the V2Suit system concept were explored and simulated prior to developing a brassboard prototype for technology demonstration. This included a system architecture for identifying the key components and their interconnects, initial identification of key human-system integration challenges, development of a simulation architecture for CMG selection and parameter sizing, and the detailed mechanical design and fabrication of a module. The brassboard prototype demonstrates closed-loop control from “down” initialization through CMG actuation, and provides a research platform for human performance evaluations to mitigate sensorimotor adaptation, as well as a tool for determining the performance requirements when used as a musculoskeletal deconditioning countermeasure. This type of countermeasure system also has Earth benefits, particularly in gait or movement stabilization and rehabilitation.

## Introduction

Exposure to the weightless environment of spaceflight is known to result in sensorimotor adaptation and physiological de-conditioning that includes spatial disorientation, space motion sickness, and significant reductions in muscle volume, muscle strength, and bone mineral density (NSBRI, 2015). Many astronauts report that the effects related to sensorimotor adaptation are the most obvious and prevalent (NSBRI, 2009). These effects are also typically most apparent during time critical maneuvering phases of a mission, just when physical and cognitive performance must be optimal to ensure mission safety and success. Launch, rendezvous and docking with orbiting platforms or bodies, and return to a gravitational environment requires precise, time-critical interactions with complex vehicle systems. In addition, emergency scenarios such as egress from a vehicle following landing require precision movements and complex coordination among the body segments to quickly and safely exit. Countermeasure systems and protocols are needed to ensure that astronauts have the required sensorimotor and physical performance capabilities to complete these critical operations during and following long-duration space missions

The “Variable Vector Countermeasure Suit (V2Suit) for Space Habitation and Exploration” is an integrated countermeasure platform designed to mitigate the spaceflight-induced physiologic adaptation and deconditioning that manifests during long-duration spaceflight and gravitational transitions. The V2Suit integrates control moment gyroscopes (CMGs) and inertial measurement units (IMUs) within wearable modules placed on multiple body segments, and when commanded in a coordinated manner they provide a “viscous resistance” against movements. This type of integrated countermeasure platform will assure astronaut health and enable safe operations, reduce the amount of time that is required for astronauts to engage in activities to mitigate these physiologic changes (Coolahan et al., 2004; Buckey, 2006), facilitate the adaptation to other gravitational environments, and reduce the mass and volume requirements for exercise equipment in future spacecraft by augmenting them with the capabilities provided by the V2Suit.

As a countermeasure system, the initial implementation of the V2Suit is envisioned to target sensorimotor adaptation, with subsequent versions targeting the musculoskeletal system. The V2Suit modules provide a whole-body coordinated resistance to movement through CMG actuation—all within an array of small, wearable, and unobtrusive modules placed on several upper- and lower-body segments and are commanded in response to body movements. In microgravity there is often no obvious up or down direction, and multiple people in the same working or living volume may be oriented differently or may perceive down differently based on the task which they are performing at the time. This “down” direction is set at the beginning of each V2Suit operational session, and when body movements are made against that direction the appropriate CMGs are actuated to provide the specified resistance which is perceived as an orientation cue. Conversely, when the body movements are perpendicular to “down” in the microgravity environment, they do not have any resistance associated with them. (This differs from movements made in a gravitational environment, which have a force acting on them regardless of the direction of movement in relation to the gravity vector). Thus, tracking the module orientation and motion is critical for the V2Suit operation. The CMGs—which generate a torque through changing the direction of the angular momentum vector (Hibbeler, 1998)—are commanded based on knowledge of their orientation and motion with respect to an initialized direction of “down.” Gyroscopic torque has been previously shown to be able to affect biomechanics by perturbing arm motions (Flanders et al., 2003), and the goal of the V2Suit is to generate a perceptible resistance to act as an additional input to the sensorimotor system. This will provide a cue to the direction of “down” and thus the wearer's orientation with respect to the initialized reference frame.

This paper describes the V2Suit system components, including the system architecture, human-system integration challenges, wearable CMG design, and the CMG control laws and navigation algorithms to track the body motions and generate a gyroscopic torque that results in the perceived “viscous resistance” during movements. Six degree-of-freedom simulations and parameterized trade studies describe the magnitude and direction of the gyroscopic torque in varying configurations and architectures. A brassboard prototype is detailed which summarizes the mechanical design and integration of the components to demonstrate the capability in a laboratory environment, and also provides a capability for subsequent testing and evaluation. The V2Suit system and simulated performance is discussed in context of its use during long-durations spaceflight as well as potential benefits on Earth.

## Background

### Spaceflight-related physiologic adaptation

All long-duration human space missions result in physiologic changes that include, but are not limited to, bone loss, muscle atrophy, cardiovascular alterations, sensorimotor adaptation (NSBRI, 2015), and the recent identification of potential changes to the visual system (NASA-HRP, 2012). Crewmembers on the International Space Station (ISS) spend approximately 2.5 h per day exercising in an attempt to mitigate this physiological deconditioning, but have not been completely successful (Coolahan et al., 2004; Buckey, 2006). Changes to the sensorimotor system typically manifest themselves during gravitational transitions and during post-flight activities, which can be observed in terms of postural instability (Paloski et al., 1999) and gait ataxia (Bloomberg et al., 2001; Bloomberg and Mulavara, 2003). Results from spaceflight suggest that when astronauts enter weightlessness, arm movements are altered and may be inappropriate and inaccurate (Johnson et al., 1975; Gazenko et al., 1982; Nicogossian et al., 1989; Paloski et al., 2008) with increased movement variability, reaction time, and duration (Bock et al., 2003). Changes in neuromuscular function (e.g., muscle fiber changes, activation potential changes), muscle atrophy, and orthostatic intolerance may also contribute to post-flight posture and stability. The sensorimotor system, however, does recover rapidly. There is an initial rapid re-adaptation period that has a time constant on the order of 2.7 h, whereas the slower, secondary, re-adaptation phase shows a time constant of approximately 100 h (4 days) (Paloski et al., 1999). On Earth, it has been shown that precision touch cues (<1 N) on the hand reduce postural sway and increase stability of treadmill locomotion (Lackner and Dizio, 2007)—a hypothesized countermeasure for sensorimotor adaptation during spaceflight. Even though the sensorimotor system appears to re-adapt rather quickly when returning to Earth, many critical tasks must occur during the gravitational transition (e.g., piloting tasks) or immediately following it (e.g., landing, vehicle egress).

Vision plays a critical role in maintaining spatial orientation in weightlessness (Oman, 2003), as well as to the performance during goal-directed arm movements (Di Cesare et al., 2014). In space, the semi-circular canals and vision continue to provide accurate information, but the otoliths no longer have a tonic input signaling gravity or body tilt, and the feet are rarely in contact with a surface. Cumulatively, this results in a conflict between the senses. During spaceflight, one of the perceptions that can change dramatically is “one's perception of static orientation with respect to the cabin and the environment beyond” (p. 376) (Oman, 2003). This altered perception can manifest as 0-G inversion illusions (Gazenko, 1964; Oman, 1986) and visual reorientation illusions (Oman, 1986). There are no countermeasures to these illusions in weightlessness. A recent experiment found that the final pointing errors during arm movements varied as a function of the direction of the visual scene tilt (Di Cesare et al., 2014)—illustrating the importance of body orientation cues on arm movement coordination. Providing an external cue to the direction of down may alleviate them, which could have operational benefits for navigation/emergency egress as well as mental rotations and reference frame coordination during teleoperation, docking or berthing operations.

The muscular system, used for locomotion, postural control, and balance is affected by spaceflight due to the gravitational unloading, the lack of a need for balance, and changes in locomotor strategies in a weightless environment (Leblanc et al., 2000a). The major effect of microgravity on this system is muscle atrophy with an accompanying loss of peak force and power (Leblanc et al., 2000a). The greatest loss in muscle mass occurs in the lower extremities and postural muscles whereas the upper body (e.g., arms) muscles seem to remain relatively unaffected. As much as a 10% loss in the deep muscles of the lower back was reported after an 8 day shuttle mission (Leblanc et al., 1995). At the whole-muscle level, the maximum lower limb power was reduced to 67% of the preflight levels in astronauts after 31 days in space, and reduced to 45% after 180 days (Gopalakrishnan et al., 2010). Another complication occurs because muscle contractions are also a major source of bone loading. Loss of muscle strength is hypothesized to exacerbate bone loss (Rittweger et al., 2005), since the mechanical stimuli that cause an osteogenic response (Rubin and Lanyon, 1987) are caused, in part, by muscle pull which impart forces larger than body weight alone. Therefore, it is necessary to develop multi-system countermeasures that address musculoskeletal deconditioning. Bone mineral density reduction rates due to spaceflight have been reported as high as 1–2% per month in the lower spine and hip, with smaller losses in the upper body (Oganov and Schneider, 1996; Leblanc et al., 2000b; Buckey, 2006). Studies of Russian Mir cosmonauts found bone losses of up to 1.7% per month in weight bearing areas such as the spine, pelvis, and proximal femur, but no loss in the upper extremities (Leblanc et al., 2000b). Similar studies performed on ISS astronauts revealed reductions of 1 and 1.5% per month in the spine and hip, respectively.

### Countermeasure suits

Several countermeasure systems have been developed and used in an attempt to prevent muscle atrophy and strength loss during spaceflight. In addition to treadmills, cycle ergometers, and resistive exercise devices, the Russian Cosmonauts have used passive stretch garments (“Penguin Suit”) and electrical stimulation. The “Penguin Suit” has “rubber bands woven into the fabric, extending from the shoulders to the waist and from the waist to the lower extremities, to produce tension on antigravity muscles” (p. 1008) (Convertino, 1996). In a head-down bed rest study, knee and ankle resistance provided by the Penguin Suit was shown to reduce soleus muscle fiber atrophy (Ohira et al., 1999). Additionally, by combining the Penguin Suit with treadmill exercise, ISS cosmonauts were found to have smaller reductions in bone mineral density of the lumbar vertebrae compared to those who did not combine the exercises (Kozlovskaya and Grigoriev, 2004; Carvil et al., 2013). More recently, a Gravity Loading Countermeasure Skinsuit (GLCS) was prototyped and evaluated in parabolic flight (Waldie and Newman, 2011). Neither of these countermeasure suits, even if combined with other exercise devices, have been shown to be fully effective in mitigating the physiologic deconditioning of the musculoskeletal system due to individual differences and varying compliance in the prescribed protocols (Vasquez, 2014).

The GLCS, as well as the “Penguin Suit,” is an example platform for integrating the V2Suit technology to provide sensorimotor and musculoskeletal benefits. Despite these types of intravehicular suits having been developed, and to a limited extent used operationally, none have proposed to integrate countermeasures for multiple physiological systems (e.g., sensorimotor, bone, muscle). These devices also have been completely passive—not containing or requiring any electrically powered components to achieve their intended purpose. The integration and use of intermittent powered components within the V2Suit aims to improve countermeasure systems being developed as well as in-flight training systems for sensorimotor adaptation. Once developed, however, integrated testing will be required to determine the appropriate dose-response characteristics of the countermeasure system required to mitigate the adaptation observed in multiple physiologic systems during long-duration spaceflight.

### Wearable kinematic systems

Miniaturized IMUs, composed of accelerometers and gyroscopes, have enabled local sensing in small wearable devices to measure human motion, without the encumbrances of wires, heavy electronics, and permanently mounted video cameras. Kinematic measurements (e.g., body angles, body segment velocities) derived from wearable IMU sensors have been used to study the biomechanics of human motion outside of laboratory and clinical settings, such as those required when using state of the art optical motion capture systems (Brodie et al., 2008; Lapinski et al., 2009). In particular, tilt and orientation may be accurately estimated using gyroscopes, accelerometers, and complementary filtering, as has been achieved for implementation in assistive devices to improve balance (Weinberg et al., 2006). Fusion algorithms that use quaternion-based representation of orientation have been used to improve accuracy, and allow for efficient real-time operation while effectively preventing “gimbal lock”— a problem seen when Euler angles are used (Favre et al., 2006). Non-linear Kalman filters, such as the extended Kalman filter (EKF) (Brown and Hwang, 1997) and the unscented Kalman filter (UKF) (Julier and Uhlmann, 1997), represent a class of fusion algorithms that can correct for the drift exhibited by inertial sensors, while providing absolute unit estimation. Recent work has demonstrated the effectiveness of this technique for tracking orientation of the torso (Luinge and Veltink, 2005) and orientation of the hand (Sabatini, 2006). A recent study has implemented a wearable IMU and EKF to study human gait and astronaut space-suited lower-body kinematics (Young et al., 2010; Young and Newman, 2011; Kobrick et al., 2012).

### Control moment gyroscopes

A control moment gyroscope (CMG) is a special type of flywheel that takes advantage of the conservation of angular momentum (Hibbeler, 1998). CMGs consist of a spinning flywheel and one or more motorized gimbals that change the angular momentum vector (Peck, 2007; Wikipedia, 2011). Gimballing the spinning mass changes the direction of its angular momentum vector, which generates an internal torque on the system. The magnitude of the torque from a single gimbal CMG (SGCMG) is proportional to the product of the angular momentum of the spinning mass and the gimbal rate, and the direction is dependent on the gimbal rotation and flywheel spin axes. The torque, τ, from a SGCMG can be approximated from the cross product of the gimbal rate vector, $\vec{\omega }$_*g*_, and the flywheel angular momentum vector, $\vec{h}$_*r*_, where τ = −$\vec{\omega }$_*g*_ × $\vec{h}$_*r*_. This is simplified from taking the time derivative of the SGCMG angular momentum vector and is valid under the assumption that the angular velocity of the body on which the CMG is attached is small in comparison to the gimbal rate of the CMG. This body angular rate is referred to as the base rate. In the case where the base rate is non-negligible, there is an internal torque generated given by: τ_*B*_ = −$\vec{\omega }$_*B*_ × $\vec{h}$_*r*_, where $\vec{\omega }$_*B*_ is the body angular rate. Therefore, the total torque generated by a CMG is τ + τ_*B*_. In the case of the V2Suit, the control system must account for these base rate effects to ensure that the appropriate direction and magnitude of torque is applied during body movements. In the ideal scenario, the base rate torque is in the desired direction for the specified resistance torque, but this is rarely the case. It is often the case that the base rate torque must be nulled through active gimballing to prevent undesired perceptions. A single SGCMG is capable of generating a torque vector that may lie anywhere on a two-dimensional surface at a given instant. While one SGCMG may occasionally be able to generate the desired torque for an attitude control system, at least three SGCMGs are generally required to generate torque in three dimensions—this is referred to as the torque envelope. Therefore, the resultant torque is from a combination of the SGCMGs, and these groupings controlled together are referred to as CMG arrays.

## V2Suit system design and analysis

The V2Suit is a complex system concept and several aspects of the design were explored and simulated prior to developing the brassboard prototype for technology demonstration. This included the development of a system architecture for identifying the key components and their interconnects, initial identification of the key human-system integration challenges, development of a simulation architecture for CMG selection and parameter sizing, and finally the detailed mechanical design and fabrication of the module. These key areas are detailed in the following sections.

### System architecture

**Figure 1:**
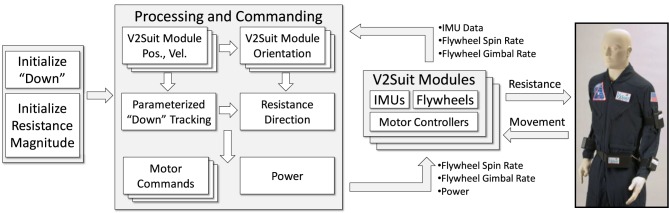
**High level V2Suit system architecture**.

At a high level, the V2Suit system is comprised of two main elements: (1) the wearable modules that are placed on multiple upper- and lower-body segments, and (2) central processing and commanding to coordinate the tracking and actuation of each module (Figure 1). Each module includes an IMU to measure its linear accelerations and angular velocities and the miniature CMG array to generate the torque with the desired direction and magnitude. Each module is connected to the central processing unit through cabling that interfaces with the wearable garments. Through this cabling, the modules receive power from the central processing and commanding module, as well as the specified flywheel spin and gimbal rates commands. Data from the IMU, flywheel spin and gimbal rates are also transmitted to the central processing and commanding module through the wearable cabling.

The central processing and commanding module contains the initialization, navigation, and control software elements. Initialization enables the parameters within the system to be specified, including the direction of “down” and the magnitude of the commanded resistance during movements that are made against “down.” The IMU data from each module is processed to determine its orientation and motion with respect to the specified coordinate system. To provide a whole-body coordinated resistance during movements, knowledge of the relative navigation state of each module with respect to one another is required. Finally, with the system initialized, and knowledge of the position, orientation, and velocity of each module, the appropriate CMG commands may be sent to each module during the operations phase to generate the appropriate torque during body movements.

### Human-system integration

The interface with the human is critically important for the operational implementation of the V2Suit. The ability of the CMGs to both generate the desired torque and apply a resistance to movements requires that each module be rigidly attached to the body (i.e., no relative motion between the module and the body segment it is attached to). This is the key to providing the coordinated “viscous response” with a specific magnitude and direction. In addition, the V2Suit must be easily put on, be comfortable to wear, and be small and low-profile as to not interfere with normal movements or activities, all while providing the desired functionality. The modules must not interfere with normal, daily activities when worn and non-operational. This requires a small form factor that can be integrated with normally worn garments—either as an add-on to existing equipment or designed as an integral part of the garment. The V2Suit module placement and interface to the human body was investigated through computer aided design (CAD) modeling, form-factor analysis using a life-size mannequin and through limited evaluations by members of the V2Suit team (Duda and Newman, 2013). The modules were sized according to the anticipated final size and shape through technology selection, component miniaturization, and packaging. They were placed near each body segment's center-of-mass (e.g., upper arm, lower arm, thigh, shin—Yeadon, 1990) in an effort to maximize the perceptual magnitudes.

### “Down” tracking algorithm

The “down” tracking algorithm is a key element of the V2Suit system. The algorithm relies on the motion sensing capabilities of the IMU, and computes the module's orientation and motion relative to the initialized “down” direction. Tracking the module's navigation state vector against this initialized direction allows the control algorithms to command the CMGs appropriately for generating the resistance to motion. The algorithm was developed and tested using both simulated data generated as well as actual IMU data collected from representative motions.

#### Initialization phase

The initialization phase is executed at the beginning of each operational session to define the direction of inertial “down,” determine the initial direction of “down” in module coordinates, and determine the initial orientation of each the V2Suit module with respect to the user-defined inertial coordinate frame (ICF). This phase is also used to tare the IMU readings. While taring, the V2Suit system software zeros the IMU acceleration and angular velocity readings to remove any biases that could affect the stability and long-term “down” tracking performance.

Since there is often no obvious “up” or “down” direction in microgravity, and multiple people in the same space may be oriented differently and perceive down differently, the V2Suit user must re-define the ICF each time the system is initialized. This ensures that the direction of “down” coincides with the desired direction for that operational session. The initialization process relies on the IMU to determine the module's direction of “down” and the initial orientation, which requires specific inputs to the system to define these parameters.

**Figure 2:**
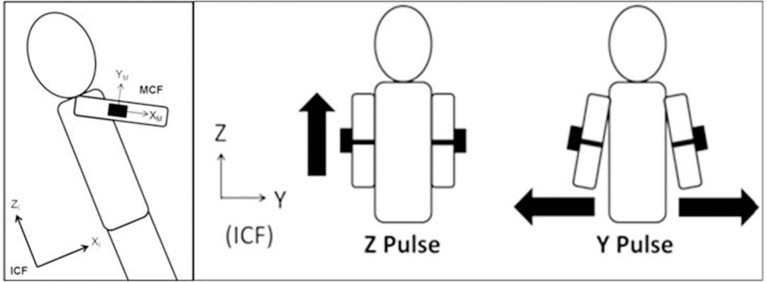
**Left:** Representation of the inertial coordinate frame vs. module coordinate frame, **Right:** The motions used to generate two acceleration pulses during initialization to define the direction of inertial down and determine the orientation of the V2Suit module with respect to the ICF. The location of the module placement on the arms in this figure is for illustration purposes only.

The initialization process requires two acceleration pulses in the wearable module axes that are parallel to two inertial axes—for example, a “Y-pulse” and a “Z-pulse” (Figure 2). One pulse is used to define the direction of “down” and the second defines the orientation of the module with respect to the ICF (Horn, 1987). On Earth, initializing “down” is trivial; “down” is simply the direction of the IMU acceleration reading due to gravity. Thus, on Earth with “down” aligned to the gravity vector the user must only generate one pulse—a “Y-pulse.” Conversely, in microgravity, the user must provide both the “Y-pulse” and “Z-pulse” motions. To specify the orientation of each individual module coordinate frame (MCF) with respect to the ICF, two symbolic coordinate systems were defined—one representing the ICF and the other representing the MCF. An orthonormalization process was used, which allows for the “Y-pulse” and “Z-pulse” to not be perfectly perpendicular (i.e., the “Y-pulse” need not lie in the inertial XY plane); however, the “Y-pulse” must be in the inertial YZ plane to get an accurate orientation. The resulting matrices are then used to calculate the rotation matrix describing the rotation between the ICF and the MCF, which was then converted to a unit quaternion.

#### Operations phase

The system is transitioned to the operations phase following a successful initialization. In this phase, the wearer moves freely with the V2Suit modules attached and the CMGs are commanded according to their orientation and movement relative to “down.” The IMU senses the angular velocity and linear acceleration of each V2Suit module in the MCF and outputs the rotation angles of the IMU relative to its zeroed state. This information is used along with the initial orientation quaternion and the initialized direction of “down” to keep track of the direction of “down” in the MCF as well as the module's position, orientation, and velocity with respect to the ICF.

The CMG-generated torque is orthogonal to the “down” vector and applied only during movements that are made against that direction. Inertial down does not change unless the system is re-initialized, but the direction of “down” in the MCF changes continuously as the module rotates during movement. This direction is tracked in order to send the appropriate inputs to the CMG controller. During the operations phase, Euler rotation angles are converted to a quaternion, *q_m_*, which describes the motion of the module. The initial “down” vector is then rotated by *q_m_* to give the direction of down in the MCF at each instant throughout the motion.

The V2Suit system will only command the CMGs to provide a resistance to motions that have a component against the initialized direction of “down.” As a result, an estimation of the linear velocity of the module is required. This velocity is estimated from the IMU linear acceleration measurements. If the module motion does not have a component in the direction of “down,” the dot product of “down” and the linear velocity vector, both in the MCF, will be equal to zero. If this dot product is non-zero, the CMGs will be commanded to generate a torque proportional to the magnitude of the linear velocity component parallel to the direction of “down;” otherwise there will be no torque commanded from the V2Suit module.

The CMG generated torque from the V2Suit module should have a perception equivalent to a gravitational torque during movements on Earth. That is, the direction of this torque should be in the same direction as a gravitational torque would be—on Earth this is during movements against down. The direction of the gravitational torque is the cross product of a unit vector starting at the joint and pointing along the body axis and a unit vector in the direction of the acceleration due to gravity. For the V2Suit, this is the module X-axis crossed with the “down” vector in MCF to specify the direction of the commanded torque, also in the MCF.

#### Algorithm performance data

Two sets of motion data were generated for development and test of the “down” tracking algorithm performance—synthetic data from a computer simulation of arm motions and actual IMU data from equivalent motions. Within each set of motion data there was a “Lift Arm” where the user starts with their arm at their side and lifts it 90° to the side, and a “90 − 90 − 90 Rotation” where the module is rotated around each MCF axis to represent a reaching activity. The algorithm development was primarily done with the computer simulation data, and then tested using the actual IMU data.

**Figure 3:**
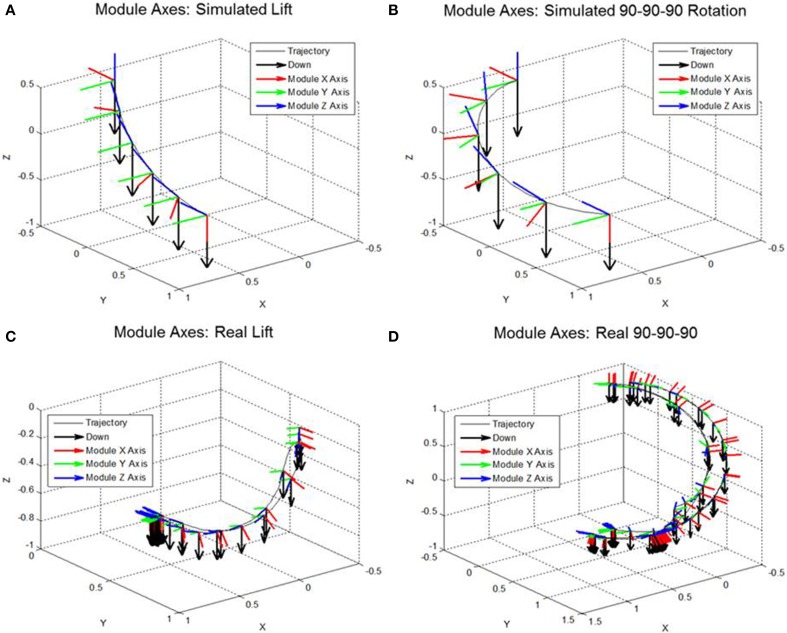
**Module Axis Tracking simulation results: (A) Simulated Lift Arm, (B) Simulated 90 − 90 − 90 Rotation, (C) Actual Lift Arm data, and (D) Actual 90-90-90 Rotation**. The module axes in the inertial coordinate frame over the course of the motion are depicted as well as the trajectory of the module and the direction of inertial down.

In the “Lift Arm” motion, the simulated user starts with their arm at their side and lifts it 90° to the side. There is only rotation about the module Y axis. The “90 − 90 − 90 Rotation” also begins with the user's arm at their side; however, the arm is simultaneously raised in the sagittal plane, rotated about its long axis, and rotated in the transverse plane. As a result, the module is rotated around each MCF axis to reach the same final position as the first motion. The computer generated IMU data (linear acceleration and angular velocity) from both of these motions were run through the “down” algorithm. Figures 3A,B show the direction of “down” in the MCF during these motions, as well as the orientation of the module with respect to “down”—overlaid on the three-dimensional trajectory of the arm motion. The same motions were repeated with an IMU attached to a wearer's arm in the approximate location of where the V2Suit module would be. This recorded representative data to pass through the algorithm. Unlike the simulated data, due to the user's movements there is some biomechanical variability in the start point, trajectory, and module orientation throughout the trajectory. The “down” tracking results from the real arm motions are shown in Figures 3C,D. As a result, the trajectory and performance plots do not exactly match the simulated data due to these biomechanical differences.

Integrated long-term stability performance of the selected IMU and the “down” tracking algorithm was also quantified. The IMU was placed in a “rest” position (flat on the table) and the reported “down” vector was recorded. It was then attached to a hand (as it would be within a V2Suit module) for 1 h as normal activities within the laboratory were performed. The IMU was returned to the rest position every 10 min and the direction of the “down” vector noted. The deviation from the original reported “down” was calculated at each rest interval. The directional offset from the original, true “down” over the course of1 h does not exceed 1° (Vasquez et al., 2014). This result provided confidence in the performance of the IMU to support long-duration operation without requiring re-initialization.

### CMG architecture and parameter sizing

Two CMG architecture and parameter sizing simulations were run. The first was determine the array of SGCMGs that would meet the V2Suit performance requirements of commanding a specified torque magnitude against the initialized direction of “down,” and the second set of simulations were run to determine the SGCMG parameters (flywheel inertia and spin rate, and gimbal rate) within the selected array to inform a detailed design. This simulation was motivated by the fact that the smallest commercially available CMGs, which are used for miniature satellite stabilization and control, are too large to be incorporated into such a wearable system. In conducting these simulations and design, two challenges associated with a miniature CMG had to be addressed simultaneously: (1) minimizing the volume while still maintaining the capability to generate the required torque, and (2) development of control algorithms that can maintain the required torque magnitude and direction. Both of these challenges must be addressed simultaneously. The analysis of various candidate CMG arrays to determine the appropriate architecture inside each V2Suit module, as well as flywheel size, flywheel spin rate, and spin assembly gimbal rate—using a candidate implementation of the control algorithms—is detailed here.

#### CMG arrays simulation and selection

**Figure 4:**
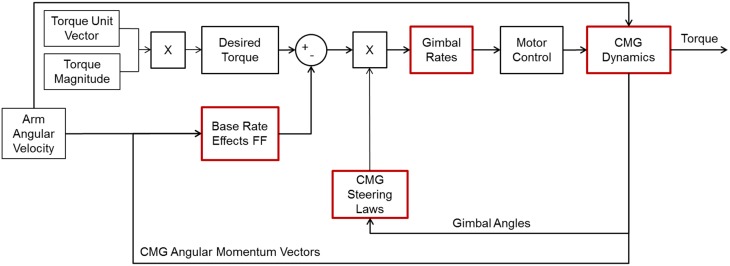
**SGCMG simulation architecture to select an array for the V2Suit**. The inputs to the simulation are the direction of the torque vector in the module coordinate frame (determined by “down” tracking), the desired torque magnitude, and the angular velocity of the arm. The red boxes indicate aspects of the simulation that are unique to each array.

Several candidate CMG architectures were simulated and evaluated during the initial architecture trade study (Vasquez, 2014). This included evaluating the ability of each of the architectures to generate at least 0.1 N-m of torque during the two simulated arm motions. This torque magnitude has been shown to provide biomechanical perturbations during reach motions (Flanders et al., 2003), and was used as an initial starting point for the V2Suit performance requirements as a sensorimotor countermeasure. The high-level simulation architecture for the SGCMGs is shown in Figure 4, where the boxes outlined in red represent aspects of the simulation that are unique to each individual CMG array. For the SGCMG architectures, the simulation uses pseudo-inverse steering laws to determine the required gimbal rate commands based on the arm motion and the desired torque magnitude and direction (Vasquez, 2014). The simulations also allow for parameterization of flywheel dimensions, material properties, spin rates, and gimbal rates. In addition to evaluating their ability to output the desired torque magnitude and direction, the architectures were also evaluated against their response characteristics in terms of spin and gimbal rates and accelerations as well as the anticipated size when packaged.

The candidate architectures evaluated included scissored pairs, pyramid, variable speed, and reaction wheels. A scissored pair is a grouping of two SGCMGs that act together to generate torque in one direction (Brown and Peck, 2008). They are gimbaled at equal and opposite rates which results in a net torque vector from the pair in a constant direction. Typically, three pairs are arranged to provide the required torque envelope as well as redundancy. Pyramid arrays consist of a group of SGCMGs arranged so that their gimbal axes are perpendicular to the faces of a pyramid with a skew angle of α (Wie, 2005), where α is typically 54.73° to create a near spherical momentum envelope for the 4-CMG array (Giffen et al., 2012). In a variable speed CMG, the flywheels are gimbaled at a constant rate and the speed of each flywheel is varied. The architecture that was considered included four sets of four flywheels attached to a single gimbal—for a total of 16 flywheels driven by four gimbal motors. The dynamics of variable speed CMGs differs from those of constant speed CMGs because the changing of both the gimbal angle and the spin rate of the CMG contribute to torque generation. Lastly, a reaction wheel array was considered where three reaction wheels were included with their spin axes aligned along one of the principle module axes. Changing the spin rate of the flywheel generates a torque aligned with the spin vector (there is no gimballing of the spin mass). Therefore, in this architecture each reaction wheel controls the torque along one axis.

In general, the simulations of the CMG arrays confirmed that a larger flywheel spinning faster will more readily generate 0.1 N-m of torque without exceeding a 60 rpm gimbal limit than a smaller flywheel spinning slower. (The 60 rpm gimbal limit was chosen based on the expected performance of candidate commercially available motors for the V2Suit). This is not a surprising result. However, the goal is to minimize both the mass of the flywheel and the spin rate, while maintaining a limit on the gimbal rate. Ultimately, the four CMG pyramid array was chosen (see Vasquez, 2014). It required the largest flywheel and fastest spin rate of the candidate arrays, but it also required the fewest number of flywheels and actuators—a key factor in minimizing the V2Suit module size. It also generated the desired torque without any concerning deviations in magnitude or direction. Subsequent simulation and refinement on key parameters within the 4-SGCMG tradespace was conducted to support component selection; this is detailed in the next section. The remaining arrays considered were not selected due to their impacts on size (e.g., 5-SGCMG array, scissored pairs) or the large spin or gimbal rates required to achieve the torque performance (e.g., reaction wheels, variable speed CMG).

#### Detailed parameter simulation and selection

**Figure 5:**
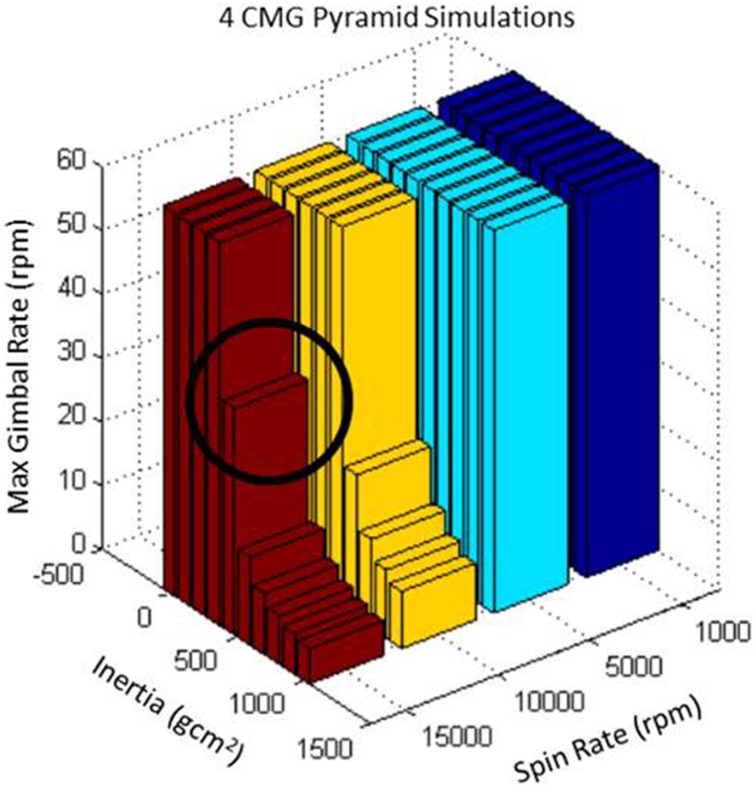
**Parametric results from the four CMG pyramid simulations**. The ideal parameters are a flywheel spin rate of 15,000 rpm with an inertia of approximately 450 g*cm^2^.

Detailed simulation and analysis of the 4-CMG array was conducted in order to select the appropriate design parameters for use in the V2Suit. As with the prior simulations, these were also run with the two simulated arm motions. The parameterized simulation, including the steering laws, was created with the goal of trading key design parameters while ensuring that the array generated the desired direction and magnitude of torque—at least 0.1 N-m in any direction. The flywheel spin rate (1000–15,000 rpm) and flywheel inertia (10^−8^–10^−4^ kg*m^2^) were both parameterized, and the flywheel gimbal rate was the free parameter. These simulations also assumed cylindrical tungsten flywheels with density 18,269 kg/m^3^. Figure 5 summarizes the results of the parameter combination; gimbal rates that exceeded 60 rpm were truncated. To function as a sensorimotor countermeasure and meet the requirements of the V2Suit wearable modules, the preferred parameters are specified by a small flywheel inertia spinning at a slow rate such that the gimbal rate does not exceed 60 rpm. For the 4 CMG pyramid array this corresponds to a flywheel of inertia approximately 450 g*cm^2^ spinning at 15,000 rpm. In subsequent versions of the V2Suit, where the system is used to target musculoskeletal deconditioning, a much larger torque magnitude will be required. This simulation will enable the identification of key sizing parameters by investigating tradeoffs in those parameters that enable the system to generate the required torque magnitude.

**Figure 6:**
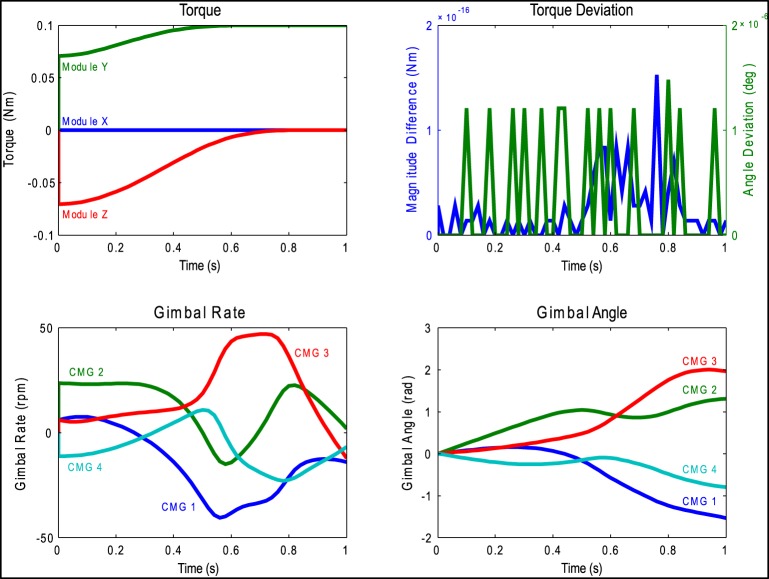
**Results from the four CMG pyramid simulation with the 90-90-90 motion for an array with steel flywheels of radius 2 cm and height 1 cm**.

With these key design parameters identified, the simulations were run again to quantitatively evaluate their performance during the “Lift Arm” and “90 − 90 − 90 Rotation,” again using the same set of pseudo-inverse steering laws. Figure 6 shows the results of the 4 CMG pyramid array during the “90 − 90 − 90 Rotation” motion, including the torque generated during time, deviation from the commanded torque, gimbal rates, and the gimbal angles for each CMG in the array. The average magnitude of torque deviation was 0.02 N-m, and there was no significant deviation in the direction of the torque vector. The magnitude deviation is due to the fact that the gimbal rates are not reached instantaneously after they are commanded; this is limited by the dynamics of the gimbal motor. (There is potential to account for the delay caused by the motor acceleration in more complex steering laws). In these simulations, the gimbal rate required to generate the desired torque never exceeded 50 rpm and the gimbal angle never exceeded one full revolution in either direction. This is beneficial since the gimbals will have a limited rotational range of motion and will need to be re-set when they reach this limit; this means that there will be periods of time during which the system is not capable of generating the desired torque. Slower gimbal rates and smaller gimbal angles mean that the CMGs can operate for longer periods of time without having to be reset. The selection of these key SGCMG design parameters enabled the subsequent detailed mechanical design and brassboard prototyping.

### Detailed mechanical design and brassboard prototype

A major design goal for the V2Suit module is to minimize the overall size so that the form factor will ultimately be wearable and unobtrusive. This project designed a CMG array with the parameters defined by the simulation analysis in an as-small-as-possible form factor using a combination of custom and off-the-shelf components. The main challenge is packaging the CMG array and associated electronics, cables, and assembly hardware into a functional and minimally sized module. Additional design considerations for the module include safety and comfort for the wearer. The 4-CMG pyramid array utilizes the fewest number of CMGs of any of the candidate arrays, which fosters miniaturization. The module needs to contain four SGCMG assemblies and the associated circuitry and electronics to control them, as well as an IMU.

#### Spin and gimbal assembly design

The V2Suit CMG spin assembly consists of a spinning flywheel mass, spin motor, an enclosure to surround the spinning mass, and the associated bearings and assembly hardware. All parts were designed with the goal of minimizing the volume of the assembly as well as the total volume of the sweep generated by revolving the assembly one full revolution around the gimbal axis. This sweep volume is a key driver of module size. The cylindrical shape for the enclosure and the design of the spinning mass are key to minimize the gimbal sweep. The flywheel spin mass was designed as a cup shape, which fits around the spin motor. This shape allows for a high ratio of the inertia of the spin mass to the total volume of the mass/motor combination. The mass is made of a high-density tungsten alloy, which gives an inertia about the spin axis of approximately 364 g*cm^2^, slightly smaller than the desired inertia. Despite the slightly smaller inertia, the pyramid array is still capable of generating the desired 0.1 N-m of output torque.

The spin assembly is canted so that the gimbal axis is at an angle of 35.27° relative to the base of the module, making it perpendicular to the face of a pyramid with an elevation angle of 54.73° (Giffen et al., 2012). The gimbal assembly has two supports for the spin assembly and the gimbal motor; a pre-loaded bearing at each face reduces the rotational friction. A cable management spool surrounds the spin assembly.

#### Integrated module design

**Figure 7:**
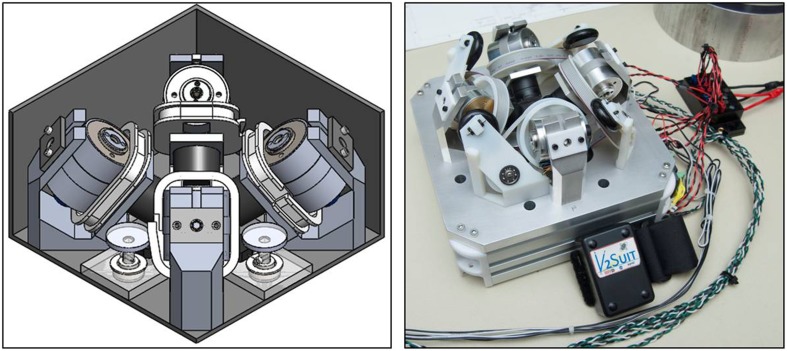
**Left:** Detailed mechanical design of a prototype V2Suit module. **Right:** As built V2Suit CMG array prototype.

The detailed design of a prototype module is shown in Figure 7. The module has a 6 inch square footprint and is 3.5 inches in height. This is larger than would be eventually planned for a body worn form factor. The constraints of leveraging commercial off-the-shelf equipment prevented this initial design from further volumetric reductions. The IMU is located in the center of the module, and the motor electronics are remote in the central power and processing blocks. The spools for cable management for each CMG are in the space adjacent to the CMGs. Orienting the CMGs into the module's corners rather than having them aligned with the module axes allows the overall size of the module to be smaller. As mentioned earlier, the gimbal sweep was taken into account in this design and, as a result specified the relative positions of the CMGs.

#### Brassboard prototype development and operations

The mechanical design for the CMG array was used to construct a prototype module (Figure 7). This prototype was developed to test the steering laws to generate the V2Suit system torque; it was not designed and built to be the ultimate wearable form-factor. The unit is connected to wall power and is commanded from a desktop computer. Module movement data, from the on-board IMU, is received by the computer and is incorporated into the steering laws which send the appropriate gimbal movement commands back to the module. Simulation and operation data indicates that this individual module design can generate a spherical torque envelope of up to 1.0 N-m, and consumes 8–10 Watts of power during operation—all using commercially available motors and motor controllers. This torque envelope is an order of magnitude greater than that hypothesized (0.1 N-m) to function as a sensorimotor countermeasure, and allows this brassboard prototype to function as a research tool for determining the appropriate resistance magnitude for targeting this physiologic system. However, to function as a musculoskeletal countermeasure system the torque magnitudes will likely need to be even greater.

This prototype can easily be held in one's hand. The unit was designed and enclosed for safe operation during initial testing and evaluation, which increases the entire volume. Future design iterations, technology research and development, and improvements in key areas such as motor technology will likely enable further reduction of the module size to a wearable form factor.

## Discussion and conclusions

The “Variable Vector Countermeasure Suit (V2Suit) for Space Habitation and Exploration” is a novel system concept that will provide a platform for integrating sensors and actuators with daily astronaut intravehicular activities to improve human health and performance, while reducing countermeasure technology mass and volume, and required exercise time during long-duration space exploration missions. The V2Suit system leverages wearable kinematic system technology and uses integrated CMGs within a miniaturized module placed on multiple upper- and lower-body segments to provide a “viscous resistance” during movements—a countermeasure to the sensorimotor and musculoskeletal adaptation performance decrements that manifest themselves while living and working in microgravity and during gravitational transitions during long-duration spaceflight, including post-flight recovery and rehabilitation.

A detailed simulation architecture was created for the development of the “down” tracking algorithm and to test its performance—a key enabler for the successful implementation of the V2Suit—as well as conduct a trade study and parameter selection of the appropriate CMG array and parameters. The “down” tracking algorithm has two modes of operation—an initialization phase where the direction of “down” is specified by the user, and then stored for the operations phase where the V2Suit system tracks the motion and orientation of each module with respect to that “down” direction. Demonstration of the algorithm during two representative arm motions (i.e., “Lift Arm” and “90 − 90 − 90 Rotation” movements) indicated satisfactory “down” tracking performance and appropriate specification of the torque vector. Additionally, with the selected IMU in the Earth-based laboratory environment, the direction of the “down” vector was maintained in the appropriate direction for up to 1 h. Further investigations into the stability of the algorithm during long-term operation will be required for longer-term operations. The simulation architecture was also used to conduct a detailed CMG trade study with the selected steering laws, and subsequent parameter selection. Several CMG architectures were evaluated, including scissored pairs, 4- and 5-CMG pyramid configurations, variable speed CMGs, and reaction wheel assemblies. Key CMG parameters such as flywheel spin rate, gimbal rate, and flywheel inertia were varied within the architecture. A 4-CMG pyramid was selected to generate the required torque of at least 0.1 N-m in any direction.

Initial design and fabrication of a 4-CMG array was conducted using commercial off-the-shelf components, and custom machining when necessary. The goal of this brassboard prototype was to demonstrate the V2Suit concept—closed loop control from “down” tracking to CMG actuation—as well as determine the key engineering and technical challenges required to overcome prior to an operational V2Suit system. The key challenges and components that require further investigation in future systems include the identification of custom electronics (motors and motor controllers), power consumption and source sizing, and an initial estimate of human-system integration options. The brassboard unit components were controlled from a desktop computer and powered from a laboratory outlet power supply. Qualitative and quantitative evaluations of the system demonstrated the ability to initialize and track against a specified direction of “down” as well as the ability to command the four CMGs independently. In addition, the single module power consumption was measured at approximately 8–10 Watts during operation.

This design specifications of the current prototype, as well as the candidate placement of the modules on the body segments, is intended to target sensorimotor adaptation by providing a low-magnitude coordinated resistance to movements made against the specified direction of “down.” The implemented brassboard prototype, which is holdable and as a spherical torque magnitude envelope of up to 1.0 N-m, provides a capability for subsequent evaluation of the modules in the context of a sensorimotor adaptation countermeasure system. Additional research and development is required to determine the module performance requirements (e.g., torque magnitude), human-system interface (e.g., extended duration wear/comfort/chafing, quantifying the relative movement between the module and the body), optimal placement of the modules on the body segments, as well as the definition of the requirements for the V2Suit to be used as a musculoskeletal deconditioning countermeasure. Also, this prototype enables a detailed engineering characterization of its performance, including response time of the torque generation in comparison to natural arm movements, power consumption for battery sizing, and the ability of the system to output the desired torque magnitude and direction during extended duration operations. Subsequently, human-in-the-loop experiments should be conducted to determine the perceptual effects of the system, including any negative perceptions due to lags in the system or artifacts resulting from nulling of the base rate effects, as well as the effect of drift/errors in the “down” vector on the perception of the direction of the commanded torque.

The successful development, integration and operation of the V2Suit will be an enabler for space exploration mission technologies, including human health and adaptation countermeasures, autonomous health monitoring, human robotic interfaces, and adaptation and operations during artificial gravity. Several key challenges, including miniature motors, slip rings, and control electronics, battery energy density and sizing, long-term performance and stability of the “down” tracking algorithm in a microgravity environment, as well as the human response to extended duration interaction with the V2Suit must be addressed before the system can be transitioned to operations. An integrated and comprehensive countermeasure system has a measurable impact on human performance following a space mission, and mass and volume savings in the spacecraft itself. This type of countermeasure suit also has Earth benefits, particularly in gait or movement stabilization for the elderly, or rehabilitating individuals—the gyroscopes could be programmed to provide a kinematic envelope of least resistance during walking. Therefore, providing tactile feedback to the appropriate biomechanical coordination either to assist in gait correction or facilitate recovery following spaceflight.

### Conflict of interest statement

The authors declare that the research was conducted in the absence of any commercial or financial relationships that could be construed as a potential conflict of interest.
